# Evaluation of left ventricular function in immunoglobulin‐resistant children with Kawasaki disease: a two‐dimensional speckle tracking echocardiography study

**DOI:** 10.1002/clc.23213

**Published:** 2019-06-07

**Authors:** Haiyong Wang, Jing Shang, Minghui Tong, Yan Song, Litao Ruan

**Affiliations:** ^1^ Department of Ultrasound Medicine The First Affiliated Hospital, Xi'an Jiaotong University Xi'an China; ^2^ Department of Ultrasound Medicine The Second Affiliated Hospital, Lanzhou University Lanzhou China

**Keywords:** intravenous immune globulin resistance, Kawasaki disease, left ventricular function, two‐dimensional speckle tracking echocardiography

## Abstract

**Background:**

Kawasaki disease (KD) patients who are unresponsive to intravenous immune globulin (IVIG) have a high occurrence of coronary artery lesions (CALs). The characteristics of left ventricular (LV) function alternation in IVIG‐resistant patients are not well‐described.

**Hypothesis:**

Two‐dimensional speckle tracking echocardiography (STE) is a useful technique that can accurately detect myocardium subclinical dysfunction in resistant patients and may assist in differentiating patients with KD at a higher risk of IVIG resistance.

**Methods:**

A consecutive sample of 50 IVIG‐resistant patients (25 males, 2.2 ± 0.9 years), 50 IVIG‐responsive patients (27 males, 2.2 ± 0.7 years) and 50 normal subjects (27 males, 2.1 ± 0.9 years) were analyzed using STE, and receiver operating characteristic curve (ROC) analysis was utilized to determine the threshold values of STE parameters associated with IVIG resistance.

**Results:**

Compared with normal children, IVIG‐resistant patients had lower global longitudinal strain (GLS) (15.82 ± 3.32 vs 20.01 ± 2.98, *P* = 0.000) and lower global circumferential strain (GCS) (16.65 ± 3.12 vs 20.11 ± 2.86, *P* = 0.042). Both GLS and GCS in IVIG‐resistant patients were significantly lower than in IVIG‐responsive patients (15.82 ± 3.32 vs 19.95 ± 3.01, 16.65 ± 3.12 vs 19.01 ± 3.00, *P* = .000, .030, respectively). ROC analysis demonstrated that the absolute values of GLS < 16.8% and GCS < 15.9% were optimal predictors of IVIG unresponsiveness (area under the curve = 0.78, 0.75; sensitivity = 0.83, 0.79; specificity = 0.69, 0.65, respectively).

**Conclusion:**

IVIG‐resistant patients presented with more severe LV systolic dysfunction compared with IVIG‐responsive patients, which may be the result of myocarditis rather than CALs. STE may be a helpful diagnostic tool that provides supportive criteria to detect KD patients at a higher risk of IVIG resistance.

## 1 INTRODUCTION

Kawasaki disease (KD) is an acute systemic vasculitis with an unknown cause that mainly affects children <5 years of age. KD is currently the leading cause of acquired heart disease in children in developed countries.^1^ The occurrence of coronary artery aneurysm can reach 25% in untreated KD patients.^2^ The most effective treatment for KD is timely intravenous immune globulin (IVIG), which can decrease the occurrence of coronary artery aneurysm from 25% to 5%^3^; however, approximately 10% to 15% of patients remain resistant to IVIG.^4^ Previous studies showed that IVIG‐resistant patients are at high risk of developing coronary artery lesions (CALs) and need an additional dose of IVIG^4^, ^5^, ^6^; therefore, early detection of such patients is extremely helpful for clinicians to adopt optimal treatment and good prognosis.

Currently, the most commonly utilized scoring systems for identifying IVIG‐resistant patients are mainly from Japan,^7^, ^8^, ^9^ but the sensitivity and specificity in these risk prediction models are low, especially in non‐Japanese populations.^10^, ^11^ In addition, echocardiographic parameters are not included in these scoring systems. Two‐dimensional speckle tracking echocardiography (STE) is a useful technique that can accurately quantify myocardial function and detect left ventricular (LV) subclinical dysfunction with high reproducibility.^12^, ^13^, ^14^ To the best of our knowledge, this is the first study using STE to assess and predict IVIG‐resistant patients. In this study, we aimed to explore the characteristics of LV systolic function of IVIG‐resistant patients and also aimed to determine whether STE can assist in identifying KD patients who are unresponsive to IVIG.

## 2 METHODS

### 2.1 Study population

In the acute phase (prior to IVIG), we recruited 50 consecutive IVIG‐resistant KD patients and 50 consecutive IVIG‐responsive KD patients who were age‐ and gender‐matched in the First Affiliated Hospital, Xi'an Jiaotong University from January 2016 through April 2018. We also enrolled 50 age‐ and gender‐matched normal children who were referred to our hospital to undergo an echocardiography examination for a murmur without evidence of cardiac abnormalities. All KD patients met the diagnostic criteria of American Heart Association.^1^ All patients in the acute phase were treated with IVIG (2 g/kg over 12 hours) and high‐dose aspirin (80 mg/kg/day divided every 6 hours). The IVIG unresponsive patients were determined to have a persistent or recrudescent fever ≥38.3°C, which occurred for more than 36 hours after the end of IVIG infusion up to 7 days after completion of the infusion. IVIG‐responsive patients were defined as patients who had a good clinical response to IVIG and did not have persistent or recrudescent fever. The IVIG‐resistant patients were further divided into the CALs subgroup or no CALs subgroup. CALs was reflected by dilation of the right coronary artery, left coronary artery and left anterior descending coronary artery, which was defined as a Z score of ≥2.5 (SD units from the coronary artery internal diameter normalized for body surface area [BSA]).^1^ Patients with structural heart disease, chronic renal disease, severe arrhythmia and insufficient image quality were all excluded. This study was approved by the research committee of the First Affiliated Hospital of Xi'an Jiaotong University, and written informed consents were obtained from the guardians of all participants.

### 2.2 Conventional echocardiogram and laboratory data

All the KD patients in the acute phase and normal children underwent transthoracic echocardiography using a commercially available ultrasonic machine (Philips EPIQ 7C, Andover, Massachusetts) equipped with a S8‐3 transducer. Both the KD patients and normal children were placed in a calm environment for the echocardiographic examination, and subjects who were uncooperative during the echocardiographic examination were sedated with oral hydrate (25‐50 mg/kg). The LV ejection fraction (LVEF) was acquired by biplane Simpson's method of disks, and LV shortening fraction was obtained according to the guideline.^15^ The LV mass (LVM) was acquired from standard M‐mode echocardiography,^16^, ^17^ and the LV mass index (LVMI) was obtained by LVM indexed to BSA. The laboratory data of all patients in the acute phase were also acquired.

### 2.3 STE analysis

The digital images of three consecutive cardiac cycles were obtained and stored for subsequent STE analysis with a commercially available software package (Qlab version 10.6, Speckle Tracking; Philips). At least 95% of myocardial wall thickness was set to cover. When the trace of the myocardial wall was unsatisfactory, a manual adjustment was used to obtain an adequate speckle tracking image. The width region of interest was set at 5 mm, and the frame rate was set between 60 and 80 frames per second. The zero‐baseline reference point was set at the onset of the QRS complex of the electrocardiogram and was referred to as the LV end‐diastole.^18^ LV longitudinal parameters were measured from apical‐3 view, apical‐2 view and apical‐4 view, after which global longitudinal strain (GLS) and global longitudinal strain rate (GLSR) were generated.^18^ The LV long axis was set to be perpendicular to the plane of the mitral annulus in the LV apical views. Circumferential parameters were measured using parasternal short‐axis views, including LV basal level, middle level, and apical level. Then, global circumferential strain (GCS) and global circumferential strain rate (GCSR) were generated. All of the measurements were performed by two observers who were blinded to the results.

### 2.4 Reproducibility

To determine intra‐observer variability, STE parameters from 20 randomly assigned patients, including GLS, GLSR, GCS, and GCSR, were reanalyzed by the same observer 2 months after the initial analysis. For inter‐observer variability, the same patients and the same cardiac cycles were analyzed by a second observer.

### 2.5 Statistical analysis

Statistical analyses were performed using SPSS 22.0 (Statistical Product and Service Solutions Company, Chicago). Mean and SD were reported for the quantitative echocardiographic data and laboratory data. The categorical data were expressed as the count and percent. The continuous variables were first assessed for normality. For between group differences, the independent‐samples t tests were used for normally distributed continuous variables, and Wilcoxon rank‐sum tests were used for non‐normally distributed continuous variables. The differences in the parameters among IVIG‐resistant group, IVIG‐responsive group and normal group were analyzed using a one‐way analysis of variance with the Student‐Newman‐Keuls test to evaluate the differences between the two groups. A *χ* ^2^ analysis was used to assess the categorical data differences. The cutoff values of STE measurements associated with IVIG unresponsiveness were determined by receiver operating characteristic curve (ROC) curve analysis. Test reliability was assessed by calculating intra‐observer variability, inter‐observer variability, and intraclass correlation coefficients (ICCs) with a 95% confidence interval.^19^ The statistical significance was defined as a *P*‐value <.05.

## 3 RESULTS

### 3.1 The characteristics of IVIG‐responsive group, IVIG‐resistant group, and normal group

The age ranges of IVIG‐resistant patients, IVIG‐responsive patients and normal children were 2.2 ± 0.9, 2.2 ± 0.7, and 2.1 ± 0.9 years, respectively. There were 25 males, 27 males, and 27 males in responsive group, resistant group, and normal group, respectively. No significant differences were found in terms of age or gender. In the IVIG‐resistant group, 10 patients with a mild or moderate CALs and two patients with aneurysm were found, whereas in the IVIG‐responsive group, only three patients with mild CALs were found.

### 3.2 Comparison of conventional echocardiographic data and STE data among the IVIG‐responsive group, IVIG‐resistant group, and normal group

**Figure 1:**
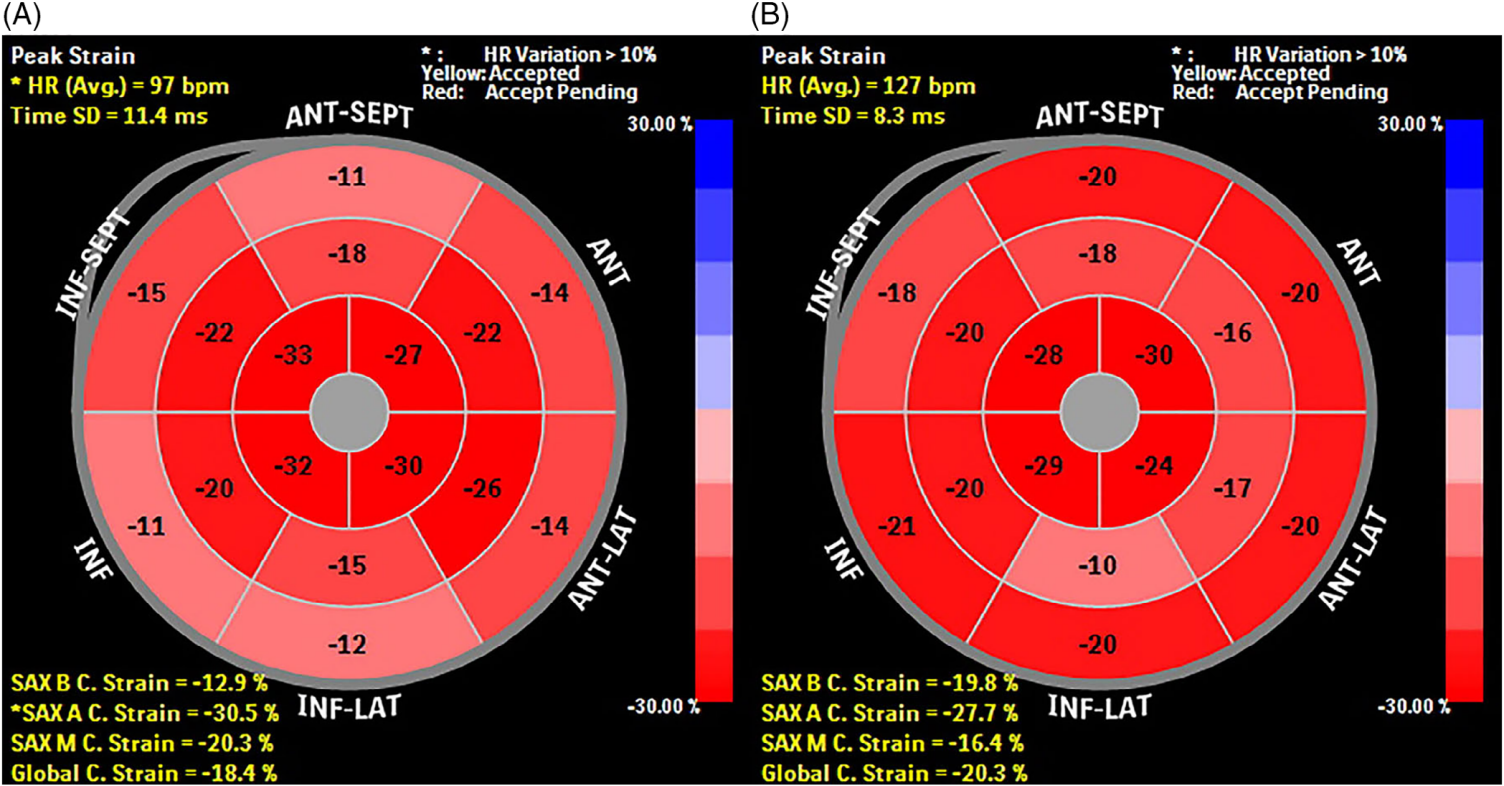
Representative examples of regional and global circumferential strain (GCS) derived from speckle tracking echocardiography (STE). A, Patient with resistant to intravenous immunology (IVIG). B, Patient with responsive to IVIG. The light red segments in A and B show the decrease in circumferential strain. HR, heart rate; Avg, average; SAX B C, short axis basal circumferential; SAX A C, short axis apical circumferential; SAX B C, short axis middle circumferential; C, circumferential

The conventional echocardiographic data and STE data are shown in Table 1. In conventional echocardiographic findings, IVIG‐resistant group had higher LVM (42.63 ± 13.81 vs 35.58 ± 14.02, *P* = .032) and higher LVMI (77.32 ± 9.91 vs 57.54 ± 8.98, *P* = .021) compared with normal group. Compared with the IVIG‐responsive group, the IVIG‐resistant group had higher occurrence of CALs (24% vs 6%, *P* = .0030), higher LVM (42.63 ± 13.81 vs 36.02 ± 14.33, *P* = .041) and higher LVMI (77.32 ± 9.91 vs 58.28 ± 9.26, *P* = .032). In STE analysis, IVIG‐resistant group had lower GLS (15.82 ± 3.32 vs 20.01 ± 2.98, *P* = .000) and lower GCS (16.65 ± 3.12 vs 20.11 ± 2.86, *P* = .042) compared with normal group. In addition, the IVIG‐resistant patients exhibited significantly decreased GLS (15.82 ± 3.32 vs 19.95 ± 3.01, *P* = .000) and GCS (16.65 ± 3.12 vs 19.01 ± 3.00, *P* = .030) (Figure 1) compared with IVIG‐responsive patients; however, there were no significant differences in GLSR and GCSR.

**Table 1:** Conventional echocardiographic data and STE data of IVIG‐resistant patients, IVIG‐responsive patients, and normal subjects

Variable	Resistant group n = 50	Responder group n = 50	Normal group n = 50
Echocardiographic data			
CALs [number, (%)]	12 (24%)^a^	3 (6%)	
LVEF (%)	67.34 ± 4.10	66.58 ± 3.98	67.81 ± 4.08
LVSF (%)	35.17 ± 5.00	37.13 ± 4.74	37.65 ± 3.98
LVM (g)	42.63 ± 13.81^a^^,^ b	36.02 ± 14.33	35.58 ± 14.02
LVMI (g/m^2^)	77.32 ± 9.91^a^^,^ b	58.28 ± 9.26	57.54 ± 8.98
STE data			
GLS (%)	15.82 ± 3.32^a^^,^ b	19.95 ± 3.01	20.01 ± 2.98
GLSR (s^−1^)	1.69 ± 0.23	1.83 ± 0.21	1.89 ± 0.18
GCS (%)	16.65 ± 3.12^a^^,^ b	19.01 ± 3.00	20.11 ± 2.86
GCSR (s^−1^)	1.97 ± 0.22	2.00 ± 0.31	2.13 ± 0.25

*Note:* Data are expressed as mean ± SD for continuous variable and as count for count variable. Strain and strain rate values are presented absolute values.

Abbreviations: STE, speckle tracking echocardiography; IVIG, intravenous immunology; CALs, coronary artery lesions; LVEF, left ventricular ejection fraction; LVSF, left ventricular shortening fraction; LVM, left ventricular mass; LVMI, left ventricular mass index; GLS, global longitudinal strain; GLSR, global longitudinal strain rate; GCS, global circumferential strain; GCSR, global circumferential strain rate.

a Resistant group vs responsive group (CALs *P* = .0030, LVM *P* = .041, LVMI *P* = .032, GLS *P* = .000, and GCS *P* = .030).

b Resistant group vs normal group (LVM *P* = .032, LVMI *P* = .021, GLS *P* = .000, and GCS *P* = .042).

### 3.3 Laboratory findings

The albumin (ALB), erythrocyte sedimentation rate (ESR), and C‐reactive protein (CRP) in IVIG‐resistant group were all higher than in IVIG‐responsive group (all *P* < .05) (Table 2).

**Table 2:** Laboratory data of IVIG‐resistant patients compared with IVIG‐responsive patients

Variable	Resistant group n = 50	Responsive group n = 50	*P* value
WBC (×10^3^/L)	16.22 ± 4.13	15.87 ± 3.98	.43
ALB (g/L)	32.12 ± 0.42	28.01 ± 0.51	.040*
CRP (mg/dL)	18.52 ± 7.81	10.91 ± 7.22	.021*
ESR (mm/h)	70.31 ± 33.01	62.61 ± 22.41	.032*
PLT (×10^3^/mm^3^)	378.71 ± 149.82	384.42 ± 179.21	.08

*Note:* Data are expressed as mean ± SD.

Abbreviations: IVIG, intravenous immunology; WBC, white blood cell; ALB, albumin; CRP, C‐reactive protein; ESR, erythrocyte sedimentation rate; PLT, platelets.

* *P* < .05.

### 3.4 ROC analysis

**Figure 2:**
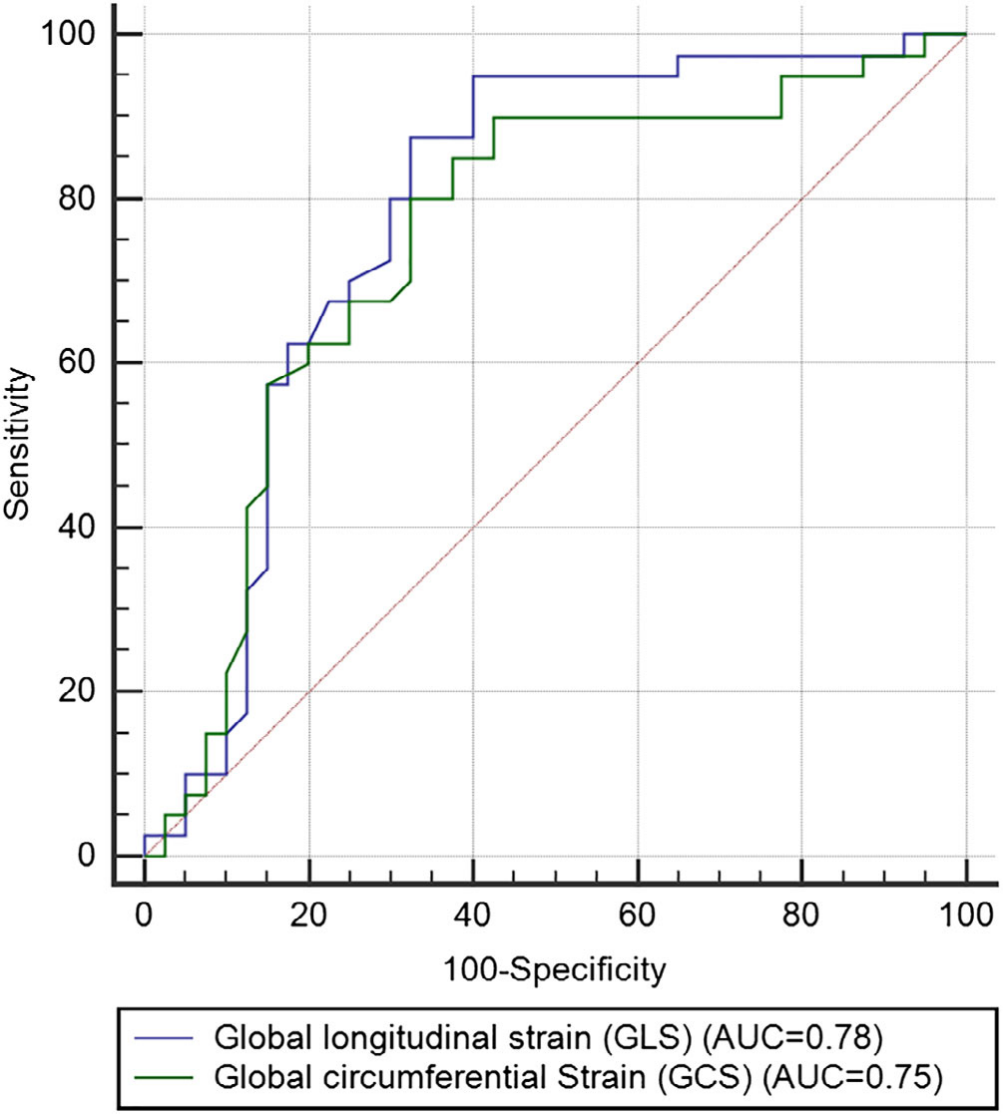
Receiver operating characteristics (ROC) curves. ROC of left ventricular peak global longitudinal strain (GLS) and global circumferential strain (GCS). AUC, area under the curve

The ROC analysis identified that the absolute values of GLS < 16.8% (AUC = 0.78, sensitivity = 0.83, specificity = 0.69, *P* = .021) and GCS < 15.9% (AUC = 0.75, sensitivity = 0.79, specificity = 0.65, *P* = .038) were reasonable predictors of IVIG resistance (Figure 2).

### 3.5 Comparison of conventional echocardiographic data and STE data between the CALs and no CALs subgroups of the IVIG‐resistant patient group

There were no significant differences in conventional echocardiographic results and STE parameters between IVIG‐resistant patients with CALs and IVIG‐resistant patients without CALs, although the occurrence of CALs in IVIG‐resistant patients was higher than in IVIG‐responsive patients (Table 3).

**Table 3:** Conventional echocardiographic data and STE data of CALs subgroup within IVIG‐resistant patients and no CALs subgroup within IVIG‐resistant patients

Variables	CALs subgroup n = 12	No CALs subgroup n = 38	*P* value
Echocardiographic data			
LVEF (%)	66.92 ± 4.13	67.80 ± 3.88	.82
LVSF (%)	36.15 ± 5.00	38.02 ± 4.67	.32
LVM (g)	43.84 ± 12.63	41.63 ± 11.87	.54
LVMI (g/m^2^)	78.43 ± 8.93	75.82 ± 9.01	.65
STE data			
GLS (%)	17.67 ± 3.12	16.58 ± 3.07	.25
GLSR (s^−1^)	1.57 ± 0.19	1.64 ± 0.23	.07
GCS (%)	18.87 ± 3.45	19.69 ± 3.18	.31
GCSR (s^−1^)	1.96 ± 0.15	2.12 ± 0.29	.052

*Note:* Data are expressed as mean ± SD. Strain and strain rate values are presented absolute values.

Abbreviations: STE, speckle tracking echocardiography; IVIG, intravenous immunology; CALs, coronary artery lesions; LVEF, left ventricular ejection fraction; LVSF, left ventricular shortening fraction; LVM, left ventricular mass; LVMI, left ventricular mass index; GLS, global longitudinal strain; GLSR, global longitudinal strain rate; GCS, global circumferential strain; GCSR, global circumferential strain rate.

### 3.6 Reproducibility

The ICCs for inter‐observer concordance was 0.962 (0.907‐0.977) for GLS, 0.988 (0.969‐0.995) for GLSR, 0.786 (0.547‐0.890) for GCS and 0.995 (0.993‐0.999) for GCSR, respectively. ICCs intra‐observer concordance was 0.973 (0.896‐0.986) for GLS, 0.871 (0.664‐0.956) for GLSR, 0.960 (0.901‐0.985) for GCS, and 0.896 (0.752‐0.965) for GCSR, respectively. Both the inter‐observer and intra‐observer concordances were good for GLS, GLSR, GCS and GCSR values.

## 4 DISCUSSION

Our study showed that IVIG‐resistant patients had both high occurrence of CALs and more serious LV systolic dysfunction than IVIG‐responsive patients. Additionally, STE may assist in differentiating patients with KD at a higher risk of IVIG resistance.

Histologic evidence of diffuse myocarditis in the acute phase of KD is present in almost all patients^20^, ^21^; however, the characteristics of myocarditis in IVIG‐resistant patients and IVIG‐responsive patients were not assessed in these autopsy studies. In our study, we demonstrated that IVIG‐resistant KD patients had both significantly lower GLS and lower GCS compared to IVIG‐responsive patients and normal children, despite the differences in LVEF and LVSF in these groups not reaching statistical significance; however, we did not find any difference in conventional echocardiographic parameters and STE parameters between responsive patients and normal children, and these findings suggested that the myocarditis in IVIG‐resistant KD patients may be more serious than in IVIG‐responsive patients. Additionally, STE is a useful technique for detecting LV subclinical systolic dysfunction in KD patients. In a study conducted by McCandless et al^22^ that used velocity vector imaging (VVI) analysis to evaluate 32 KD patients (14 patients with IVIG resistance and 17 patients with CALs) and 22 control subjects, it was found that GLS and GCS in IVIG‐resistant patients significantly decreased compared with normal subjects and our study was in accordance with such findings. However, there was no difference in GLS and GCS between resistant patients and responsive patients in their research. The reasons for this inconsistency with our study may be as follows. (a) Only the single apical‐4 chamber view and one short‐axis view in their study were used to generate GLS and GCS respectively, which may lack representativeness for whole LV function. (b) The sample volumes for both KD patients and normal subjects were small. (c) They used the VVI (Siemens Medical Solutions USA, Inc.) myocardial deformation imaging technique, and the algorithm for strain and strain rate may be inconsistent from different vendor platforms. Cardiac myocarditis in KD patients can be manifested by LV dysfunction, tachycardia and arrhythmia, and myocardial fibrosis resulting from diffuse myocarditis is the main reason for LV dysfunction in KD patients in the acute phase.^23^ LV systolic dysfunction of KD patients in the acute phase is generally transient and responds rapidly to anti‐inflammatory treatment.^24^ A study by Phadke et al^25^ used tissue Doppler imaging (TDI) to evaluate the LV function of IVIG‐resistant KD patients, and the results demonstrated that IVIG‐resistant patients had lower septal and lateral LV peak early diastolic tissue Doppler velocity of the mitral annulus compared to the IVIG‐responsive group; however, they did not find any significant difference in systolic velocity. The reason for the lack of a difference in LV systolic function may be that TDI is angle‐dependent and has a disadvantage in that only regional function represents global LV function. The systolic strain rate was regarded to be a more robust parameter reflecting myocardial function than strain^26^; however, we did not find any differences in both GLSR and GCSR among resistant patients, responsive patients and normal children, suggesting that the strain rate may not be superior to strain when assessing LV systolic function of IVIG‐resistant patients in the acute phase.

Second, myocardial swelling resulting from myocarditis and increased small vessel permeability as a result of coronary arteritis are the main pathophysiology factors of KD patients in the acute phase^21^, ^27^ leading to an increase of LVM and LVMI. Yu et al^28^ retrospectively analyzed 66 children with KD and found that KD patients in the acute phase had significantly higher LVM and LVMI compared to normal controls. In addition, the peak E velocity was related to LVMI (*r* = 0.25, *P* = .0479), suggesting that LVMI is a potential predictor of LV diastolic function and should be added to cardiac function evaluation of KD patients in the acute and subacute phase. In our study, we found that the LVM and LVMI in IVIG‐resistant patients in the acute phase were higher than those in IVIG‐responsive patients and normal children, which indicated that a greater decrease in GLS and GCS in resistant patients compared to responders may be the result of an LV geometry change due to the increased LVM. Therefore, LVM and LVMI should also be regarded as potential predictors of LV systolic function in KD patients. In our study, the inflammatory parameters, including ALB, PLT, ESR, and CRP, in unresponsive patients were all higher than in responders, which also suggested that the myocarditis in IVIG‐resistant patients may be more serious than in IVIG‐responsive patients.

Third, in our study, we found that the impaired systolic function in IVIG‐resistant patients may be due to myocardial inflammation rather than the result of coronary arteritis. IVIG‐resistant group in our research had a higher occurrence of CALs than IVIG‐responsive group, which was similar to a previous study^25^; however, there were no significant differences in systolic parameters, including LVEF, LVSF and STE parameters, between the CALs subgroup of the IVIG‐resistant patients and no CALs subgroup of the IVIG‐resistant patients. A study of 29 autopsied KD patients in the acute phase study conducted by Harada et al^21^ showed that the myocarditis of KD patients in the acute phase developed earlier than coronary arteritis. In addition, they demonstrated that severe myocarditis can manifest without any relationship to CALs. Our finding was in accordance with this autopsy result.

Finally, another finding in our study was that STE may be a helpful technique in detecting KD patients with IVIG resistance. IVIG‐resistant patients have a greater probability of CALs occurrence, and the complications of CALs, including coronary artery stenosis, thrombosis, myocardial ischemia, myocardial infarction, and even sudden death, can occur in the acute phase.^1^ Therefore, early detection of IVIG resistance is especially helpful for clinicians, allowing them to change the treatment time, decrease the occurrence of CALs, and improve patient prognosis. In an analysis of a Korean nationwide multicenter survey including 5151 KD patients performed by Kim,^29^ it was found that elevated levels of polymorphonuclear neutrophils, serum N‐terminal pro‐brain natriuretic peptide, CRP, aspartate aminotransferase, and alanine aminotransferase were significantly associated with IVIG resistance in patients with KD. In another study conducted by Ashouri et al,^30^ the results showed that an elevated band count, low ALB level, and an abnormal coronary artery can also be useful tools to identify patients at risk for IVIG resistance. Our ROC analysis results demonstrated that the GLS and GCS findings derived from STE were correlated with IVIG resistance with both high sensitivity and specificity, and resistance to IVIG therapy might be accompanied by more decreased myocardial function. Interestingly, these previous studies were mainly focused on laboratory data, and the echocardiographic data were not included. Our study results suggested that STE may help provide supportive criteria for clinicians to identify IVIG‐resistant patients at an earlier time, allowing them to adopt an optimal treatment for preventing the occurrence of CALs. Additionally, GLS and GCS should be considered for inclusion in the scoring system for IVIG unresponsiveness, and prospective cohort study with multicenter is recommended to validate the usefulness of STE in predicting patients with IVIG resistance.

This study had several limitations. First, we only assessed KD patients in the acute phase; thus, further studies with a long‐term follow‐up period are recommended. Second, different vendors are not entirely consistent regarding the algorithm of STE, which may restrict comparison among these vendors. Finally, this was only a single center study; therefore multicenter studies are recommended to verify the results.

## 5 CONCLUSION

IVIG‐resistant KD patients had more severe LV systolic dysfunction than patients who were responsive to IVIG. STE is a useful tool for detecting subclinical LV dysfunction in IVIG‐resistant patients. GLS and GCS derived from STE may be helpful diagnostic parameters that provide supportive criteria to detect KD patients at a higher risk of IVIG resistance.

## CONFLICT OF INTEREST

The authors declare no potential conflict of interests.
